# Ionotropic receptors mediate nitrogenous waste avoidance in *Drosophila melanogaster*

**DOI:** 10.1038/s42003-021-02799-3

**Published:** 2021-11-12

**Authors:** Subash Dhakal, Jiun Sang, Binod Aryal, Youngseok Lee

**Affiliations:** 1 Department of Bio and Fermentation Convergence Technology, Kookmin University, Seoul, 02707 Republic of Korea; 2 Interdisciplinary Program for Bio-Health Convergence, Kookmin University, Seoul, 02707 Republic of Korea

**Keywords:** Cellular neuroscience, Feeding behaviour, Behavioural genetics

## Abstract

Ammonia and its amine-containing derivatives are widely found in natural decomposition byproducts. Here, we conducted biased chemoreceptor screening to investigate the mechanisms by which different concentrations of ammonium salt, urea, and putrescine in rotten fruits affect feeding and oviposition behavior. We identified three ionotropic receptors, including the two broadly required IR25a and IR76b receptors, as well as the narrowly tuned IR51b receptor. These three IRs were fundamental in eliciting avoidance against nitrogenous waste products, which is mediated by bitter-sensing gustatory receptor neurons (GRNs). The aversion of nitrogenous wastes was evaluated by the cellular requirement by expressing Kir2.1 and behavioral recoveries of the mutants in bitter-sensing GRNs. Furthermore, by conducting electrophysiology assays, we confirmed that ammonia compounds are aversive in taste as they directly activated bitter-sensing GRNs. Therefore, our findings provide insights into the ecological roles of IRs as a means to detect and avoid toxic nitrogenous waste products in nature.

## Abstract (web summary)

Dhakal et al. use chemoreceptor screening on three ionotropic receptors in Drosophila, IR25a, IR76b, IR51b to evaluate their impact on avoidance behaviors against nitrogenous waste products. The results of electrophysiology assays show that ammonia compounds are aversive in taste by directly activating bitter-sensing gustatory receptor neurons.

## Introduction

Nitrogen is an essential building block for the synthesis of DNA and protein and is the most abundant element in the Earth’s atmosphere. Therefore, the nitrogen cycle is instrumental in maintaining healthy ecosystem dynamics. Animals and many plants produce nitrogenous wastes throughout their life histories. Ammonia and urea are the decomposition byproducts of protein^1^, whereas rotten fruits are rich in polyamines such as putrescine^2–4^. These organic compounds can be recycled as a source of nitrogen groups such as amines, amides, and anilines, which are central for developmental and physiological processes in both plants and animals^5^. In nature, ammonia concentration is in the range of 10–20 mM in the cassava plant leaf and the cattle manure^6,7^.

Animals, including insects, rely on chemoreception for feeding, mating, and escaping from predators^8–11^. Moreover, although the anatomy and molecular basis of taste perception in vertebrates and invertebrates are evolutionarily distinct, they share a few similar fundamentals^12–14^. Fruit flies (*Drosophila melanogaster*) possess specialized taste neurons on their labella, legs, pharynx, wing margins, and ovipositor^15–17^. Flies can sense sweetness, bitterness, sourness, saltiness, and water^18–22^. Major gustatory organs, such as the labellum and legs, have evolved to recognize chemicals via several channels and receptors, such as gustatory receptors (GRs), odorant receptors (ORs), ionotropic receptors (IRs), transient receptor potential (TRP) channels, and pickpocket ion channels (PPKs)^23–28^. Most taste sensilla harbor four distinct GRNs, of which two are attractive GRNs (sweet-sensing and water-sensing GRNs) and two are aversive GRNs (bitter-sensing or calcium-sensing GRNs)^18,29–31^. Moreover, these neuronal circuits have been found to be distinct, as attractive or aversive GRNs can be independently activated and behaviorally controlled by artificially expressing temperature-activated TRPA1, capsaicin-activated rat TRPV1, or light-activated channelrhodopsin-2^29,32,33^.

Among these chemoreceptors, IRs are broadly expressed in the peripheral sensory systems involved in chemosensation, thermosensation, and hygrosensation^16,23,34,35^. Recent studies on the mechanisms of taste perception indicate that saltiness, sourness, amino acids, and other chemical cues are directly sensed by taste IRs^15,20,36,37^. In nature, amine-containing compounds not only elicit aversive responses but have also been identified as important kairomones in host-seeking insects. For instance, insects and some disease vectors are attracted by the odor of ammonia and amines, which are excreted through sweat^38–41^. Ammonia and putrescine are olfactory cues for many anthropophilic insects, including Anopheles mosquitoes^41^. However, high concentrations of ammonia and urea have been found to dramatically decrease the fecundity and egg viability of *Drosophila suzukii*^42^. The production of urea in insects has been attributed to the catalysis of arginine hydrolysis^43^. In contrast, blood-feeding insects such as *Aedes aegypti* can efficiently detoxify ammonia-containing compounds. Specific mechanisms have evolved to tightly regulate the synthesis and excretion of nitrogenous waste and avoid the toxic effects that may result from abnormally high ammonia concentrations in tissues^44^.

The above-described studies highlight the critical role of nitrogen-containing compound perception in insects, in addition to the more widely characterized perception of sweetness, sourness, saltiness, bitterness, and water. Previous studies have investigated the mechanisms of ammonia and amino group olfactory perception in Drosophila antennae and other insects^38^; however, the cellular and molecular mechanisms that enable the taste perception of these compounds remain largely uncharacterized. Here, we elucidated the gustatory mechanisms by which Drosophila (fruit flies) sense ammonia and its derivatives in plant- and animal-derived decay products. Using a combined behavioral and electrophysiology approach, we discovered that flies perceive and avoid ammonia and its derivatives as bitter tastants via bitter-sensing GRNs. We also elucidated molecular sensors that are required for the gustatory perception of naturally occurring nitrogenous waste products.

## Results and discussion

**Fig. 1:**
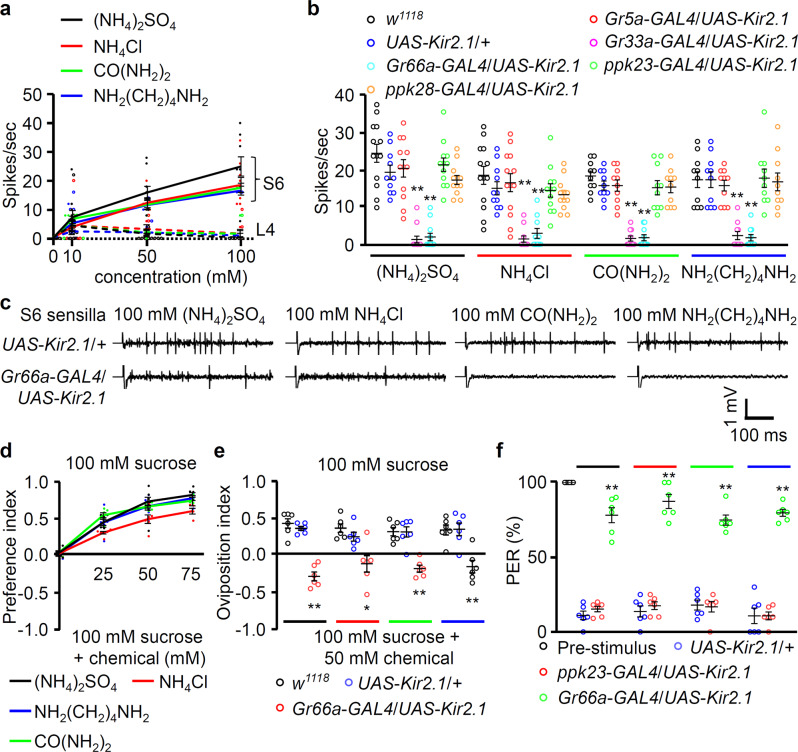
Flies avoid feeding and laying eggs on ammonia-, urea-, and putrescine-containing media via bitter-sensing GRNs. **a** Tip recording performed on the taste sensilla (solid colored lines are S6 and dashed colored lines are L4) from the labella of control (*w*^*1118*^) flies with indicated concentrations of ammonium sulfate [(NH_4_)_2_SO_4_], ammonium chloride (NH_4_Cl), urea [CO(NH_2_)_2_], and putrescine [NH_2_(CH_2_)_4_NH_2_] (*n* = 10). **b** Frequencies of the action potentials elicited from S6 sensilla using 100 mM of the indicated chemicals in control (*w*^*1118*^), *UAS*-*Kir2.1*/+, and each neuron-ablated fly, including *Gr5a*-*GAL4*, *Gr33a*-*GAL4*, *Gr66a*-*GAL4*, *ppk23*-*GAL4*, and *ppk28*-*GAL4* with *UAS*-*Kir2.1* (*n* = 10–13). **c** Representative sample traces from panel (**b**). **d** Two-way choice feeding assay showing the preferences of control (*w*^*1118*^) flies upon presentation of 100 mM sucrose alone versus 100 mM sucrose laced with the indicated concentrations of (NH_4_)_2_SO_4_, NH_4_Cl, CO(NH_2_)_2_, and NH_2_(CH_2_)_4_NH_2_ (*n* = 6). **e** Ovipositional preference assay of control (*w*^*1118*^), *UAS*-*Kir2.1*/+, and bitter-sensing GRN-ablated flies (*Gr66a*-*GAL4*/*UAS*-*Kir2.1*) between 100 mM sucrose food and food containing 100 mM sucrose laced with 50 mM (NH_4_)_2_SO_4_, NH_4_Cl, CO(NH_2_)_2_, and NH_2_(CH_2_)_4_NH_2_, respectively (*n* = 6). The line colors indicate each chemical, as in panel (**a**). **f** Average percentage of flies exhibiting the proboscis extension response (PER) after applying 100 mM of the indicated chemical stimulus to the labellum of the control (*w*^*1118*^), *UAS*-*Kir2.1*/*+*, and two aversive GRN-ablated flies (*ppk23*-*GAL4*/*UAS*-*Kir2.1* and *Gr66a*-*GAL4*/*UAS*-*Kir2.1*). A pretest was conducted using 100 mM sucrose alone. The taste stimuli consisted of 100 mM sucrose and 100 mM of (NH_4_)_2_SO_4_, NH_4_Cl, CO(NH_2_)_2_, and NH_2_(CH_2_)_4_NH_2_, respectively (*n* = 6). All error bars represent the SEMs. Multiple comparisons were conducted using single-factor ANOVA coupled with Scheffe’s post hoc test. Asterisks indicate statistical significance compared with the control (^*^P < 0.05, ^**^P < 0.01).

The perception of ammonia as an olfaction cue has been investigated in flies and mosquitoes^23,45,46^. However, only a few studies have assessed the gustatory mechanisms of ammonia perception^21,47^. The labellum is the major taste organ in *D.* *melanogaster* and possesses 31 taste sensilla in each hemisphere^16^. Each sensillum is named according to its length and position (“L4,” “I8,” “S6” indicate “long 4,” “intermediate 8,” and “short 6,” respectively). All sensilla have one sweet gustatory receptor neuron (GRN), but only S-type and I-type sensilla have bitter-sensing GRNs^16^. We found that nitrogenous wastes such as ammonium sulfate [(NH_4_)_2_SO_4_], ammonium chloride (NH_4_Cl), urea, and putrescine can activate S6 sensilla, but not L4, in a dose-dependent manner (Fig. 1a). Moreover, ammonium sulfate and ammonium chloride strongly activate S5, S6, and S7 sensilla but not L- or I-type sensilla^38^ (Supplementary Fig. 1a). The positive and negative control responses for L4 were measured with sucrose and caffeine (Supplementary Fig. 1b, c). The humidified materials of nitrogenous wastes are in the range of 10–20 mM, but we believe that these nitrogenous wastes can be easily dried to generate much higher concentration in the wild condition. GRNs are mainly classified into four different categories^16^. These GRNs include the sweet-sensing *Gr5a*-*GAL4*, bitter-sensing *Gr66a*-*GAL4* or *Gr33a*-*GAL4*, calcium-sensing *ppK23*-*GAL4*, and water-sensing *ppK28-GAL4* neurons^18,29–31,48^. Sugar and water are generally attractive, whereas bitterness and calcium are aversive. To perform an unbiased test, we expressed the inwardly rectifying potassium channel (Kir2.1) gene to inhibit each category. All four chemicals induced action potentials in the S6 sensilla of the controls (*w*^*1118*^ and *UAS*-*Kir2.1*/+) (Fig. 1a, b). These action potentials were only inhibited by the ablation of bitter-sensing GRNs (*Gr33a*-*GAL4*/*UAS*-*Kir2.1* and *Gr66a*-*GAL4*/*UAS*-*Kir2.1*), whereas the other neurons exhibited similar responses to those of the controls^6,7,49^. The results of these electrophysiological tip recordings were further confirmed by our behavioral assays (Fig. 1d–f). Flies avoided ammonium sulfate, ammonium chloride, urea, and putrescine in a dose-dependent manner (Fig. 1d). Female flies also avoided laying their eggs on surfaces containing 50 mM of each chemical, and this response was mediated by bitter-sensing GRNs (Fig. 1e). However, *Gr66a*-*GAL4*/*UAS*-*Kir2.1* female flies laid slightly more eggs on ammonia-containing food. This made us measure the pH of each chemical. Ammonium sulfate (pH 5.8), ammonium chloride (pH 5.9), and putrescine (pH 5.5) are slightly acidic in solution, whereas urea (pH 7.6) is slightly basic. To address whether the pH affected oviposition behavior and binary food choice assay, we tested ammonium sulfate which were adjusted to neutral pH by adding ammonium hydroxide (Supplementary Fig. 1d, e). The aversions of wild-type flies were not affected by the slight change of pH. Next, the slight attraction of bitter-sensing GRNs-ablated flies to the chemicals in oviposition may be affected by olfaction, because a population of OR neurons and projection neurons are activated by ammonia^40^. To test any possible roles of antenna in oviposition (Fig. 1e), we tested normal and antenna-removed female flies with the same genotypes in Fig. 1e (Supplementary Fig. 1f). We found that antenna-removed controls and *Gr66a*-*GAL4*/*UAS*-*Kir2.1* flies showed slightly increased oviposition index, but not statistically significant, compared to normal flies (Supplementary Fig. 1f). These results confirmed that the bitter-sensing GRNs in taste had a major role in affecting oviposition on the ammonia-containing media, though ammonia odor may partially influence female flies to select egg laying sites if any. Furthermore, to confirm the putative aversiveness of nitrogenous wastes and their role in activating bitter-sensing GRNs, the response of flies to each chemical was characterized via an adaptation of the proboscis extension reflex (PER) assay. This PER response was evaluated after a pre-stimulus with sucrose (Fig. 1f). As expected, this paradigm provided cellular-level evidence of the involvement of bitter-sensing GRNs, but not calcium-sensing GRNs, in the aversive response to nitrogenous waste products.

**Fig. 2:**
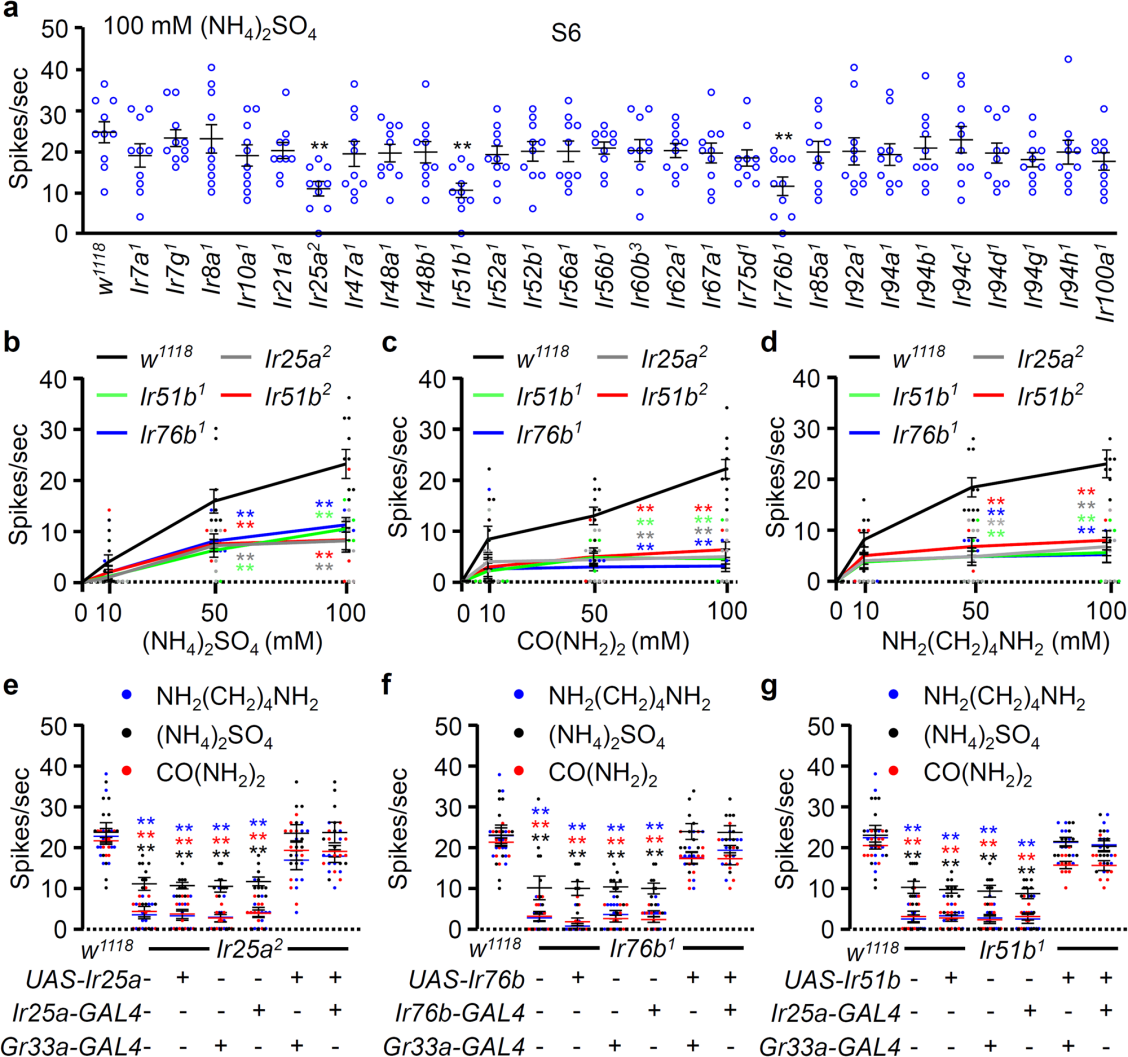
Three IRs are required for the detection of nitrogenous wastes, as determined by electrophysiology assays. **a** Average number of spikes per second when the S6 sensilla on the labellum were stimulated with 100 mM (NH_4_)_2_SO_4_ [control (*w*^*1118*^) and 28 *Ir* mutant lines] (*n* = 10). **b**–**d** Average frequencies of action potentials obtained by performing tip recordings on the S6 sensilla of the control (*w*^*1118*^) and three candidate *Ir* mutant flies in responses to the indicated doses (mM) of (**b**) ammonium salts, (**c**) CO(NH_2_)_2_, and (**d**) NH_2_(CH_2_)_4_NH_2_, respectively (*n* = 10–11). **e**–**g** Rescue of tip recording defects in (**e**) *Ir25a*^*2*^, (**f**) *Ir76b*^1^, (**g**) *Ir51b*^*1*^ mutants by expressing the respective wild-type cDNA under the control of the indicated *GAL4* promoters. Action potentials were elicited on S6 sensilla from the labellum with 100 mM of (NH_4_)_2_SO_4_, CO(NH_2_)_2_, and NH_2_(CH_2_)_4_NH_2_ (*n* = 10–12). All error bars represent the SEMs. Multiple comparisons were conducted using single-factor ANOVA coupled with Scheffe’s post hoc test. The colored asterisks in panels (**e**–**g**) indicate a significant difference between the same-colored bars compared to the control (^**^P < 0.01).

Further, we performed labellar tip recordings with 100 mM ammonium sulfate using candidate IRs to identify the molecular basis of ammonium sulfate, ammonium chloride, urea, and putrescine perception^20,23,29^ (Fig. 2a). We found deficits in three mutants of the broadly required IR25a and IR76b receptors, in addition to a newly identified IR51b mutant from 28 *Ir* mutants, which were expressed in the labellum and taste sensilla that surround the legs and wing margins (Fig. 2a and Supplementary Table 1). Tip recordings of the response of other candidate GR mutants to ammonium sulfate were conducted to further confirm the results of our screening experiments^50^ (Supplementary Fig. 2a). Once again, we found that only three IRs were required for ammonium sulfate perception. We also tested neutral ammonium sulfate for control and three IR mutants in electrophysiology (Supplementary Fig 2b). We confirmed that the pH of the ammonia had no role in the residual spikes in mutant flies. Next, tip recordings were conducted to assess the responses to the remaining chemicals. Using a 0–100 mM range, we found that the same IRs are required for sensing ammonium chloride, urea, and putrescine (Fig. 2b–d). To further support the indispensable role of these three IRs to respond to nitrogenous wastes, rescue experiments were conducted using a targeted gene expression approach via the *GAL4*/*UAS* system. These genetic experiments indicated that the deficits of *Ir25a*^*2*^ and *Ir76b*^*1*^ mutants to sense ammonium sulfate, urea, and putrescine were recovered through the expression of each wild-type cDNA under the control of the *Gr33a*-*GAL4* bitter-sensing GRNs^48^ and its respective *GAL4*, as demonstrated by our electrophysiology experiments (Fig. 2e, f). Furthermore, we rescued the *Ir51b*^*1*^ deficit by expressing *Ir51b* cDNA under the control of *Ir25a*-*GAL4* or *Gr33a*-*GAL4* (Fig. 2g).

**Fig. 3:**
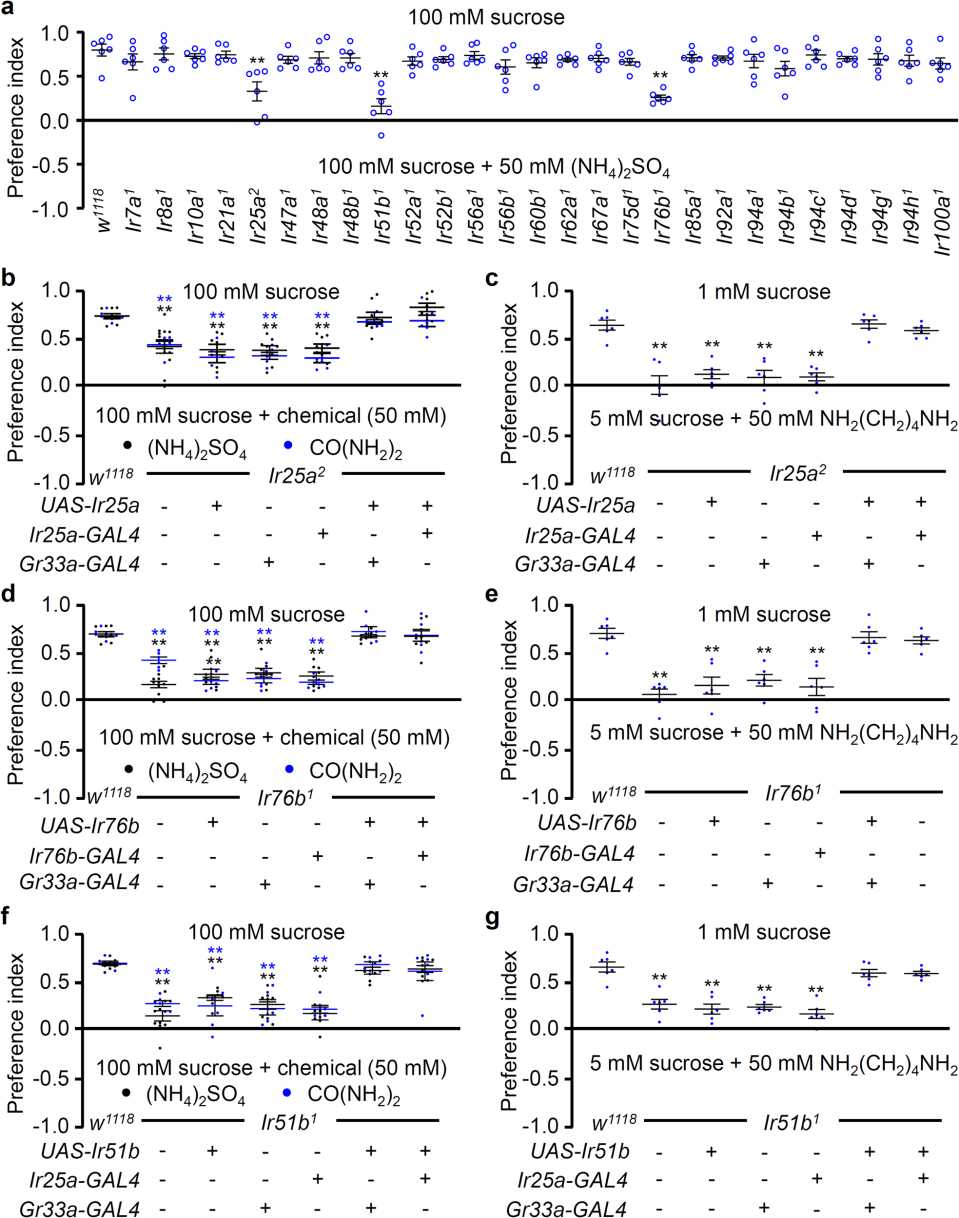
Three IRs are required to avoid toxic concentrations of nitrogenous waste products. **a** Screening of *Ir* mutants using the binary food choice assay. The flies were given a choice between 100 mM sucrose versus 100 mM sucrose laced with 50 mM (NH_4_)_2_SO_4_ (*n* = 6). **b**, **c** Binary food choice assay results showing the rescue of feeding defects in *Ir25a*^*2*^ mutants in response to (**b**) 50 mM (NH_4_)_2_SO_4_, 50 mM CO(NH_2_)_2_, and (**c**) 50 mM NH_2_(CH_2_)_4_NH_2_ when wild-type *Ir25a* cDNA was expressed under the control of *Ir25a*-*GAL4* as well as *Gr33a*-*GAL4* (*n* = 6–11). **d**, **e** Behavioral rescue of feeding defects in *Ir76b*^*1*^ mutants exposed to (**d**) 50 mM (NH_4_)_2_SO_4_, 50 mM CO(NH_2_)_2_, and (**e**) 50 mM NH_2_(CH_2_)_4_NH_2_ expressing wild-type *Ir76b* cDNA under the control of *Ir76a-GAL4* or *Gr33a*-*GAL4* (*n* = 6–11). **f**, **g** Binary food choice assays to assess the rescue the feeding defects of *Ir51b*^*1*^ mutants in response to (**f**) 50 mM (NH_4_)_2_SO_4_, 50 mM CO(NH_2_)_2_, and (**g**) 50 mM NH_2_(CH_2_)_4_NH_2_ when wild-type *Ir51b* cDNA was driven under the control of *Ir25a*-*GAL4* or *Gr33a*-*GAL4* (*n* = 6–10). All error bars represent the SEMs. Multiple comparisons were conducted using single-factor ANOVA coupled with Scheffe’s post hoc test. The colored asterisks indicate statistical significance with the same-colored bar values compared to the control (^**^P < 0.01).

To assess the molecular basis of ammonium-induced aversive behaviors, the response of the candidate IRs *Ir25a*, *Ir51b*, and *Ir76b* to a 100 mM sucrose solution versus 100 mM sucrose + 50 mM ammonium sulfate were examined via two-way choice feeding assays (Fig. 3a). The results of these behavioral assays supported the physiological evidence that mutations in *Ir25a*, *Ir51b*, and *Ir76b* only resulted in diminished avoidance to 50 mM ammonium sulfate, whereas mutation of other *Ir*s had no significant effect on ammonium sulfate avoidance in the feeding assay^40^. Three IRs may also affect olfaction, while taste is likely a main determinant of ammonia aversion in feeding behavior which is highly attributed to changes in taste physiology.

Similar to the electrophysiological responses, we confirmed that the same *Ir* mutants exhibited behavioral responses to ammonium sulfate, urea, and putrescine concentrations ranging from 0 to 75 mM (Supplementary Fig. 3a–c). Additionally, upon rescuing the deficits of these mutants via genetic experiments, the results of our behavior assays were consistent with those of our electrophysiology recordings (Fig. 3b–g).

**Fig. 4:**
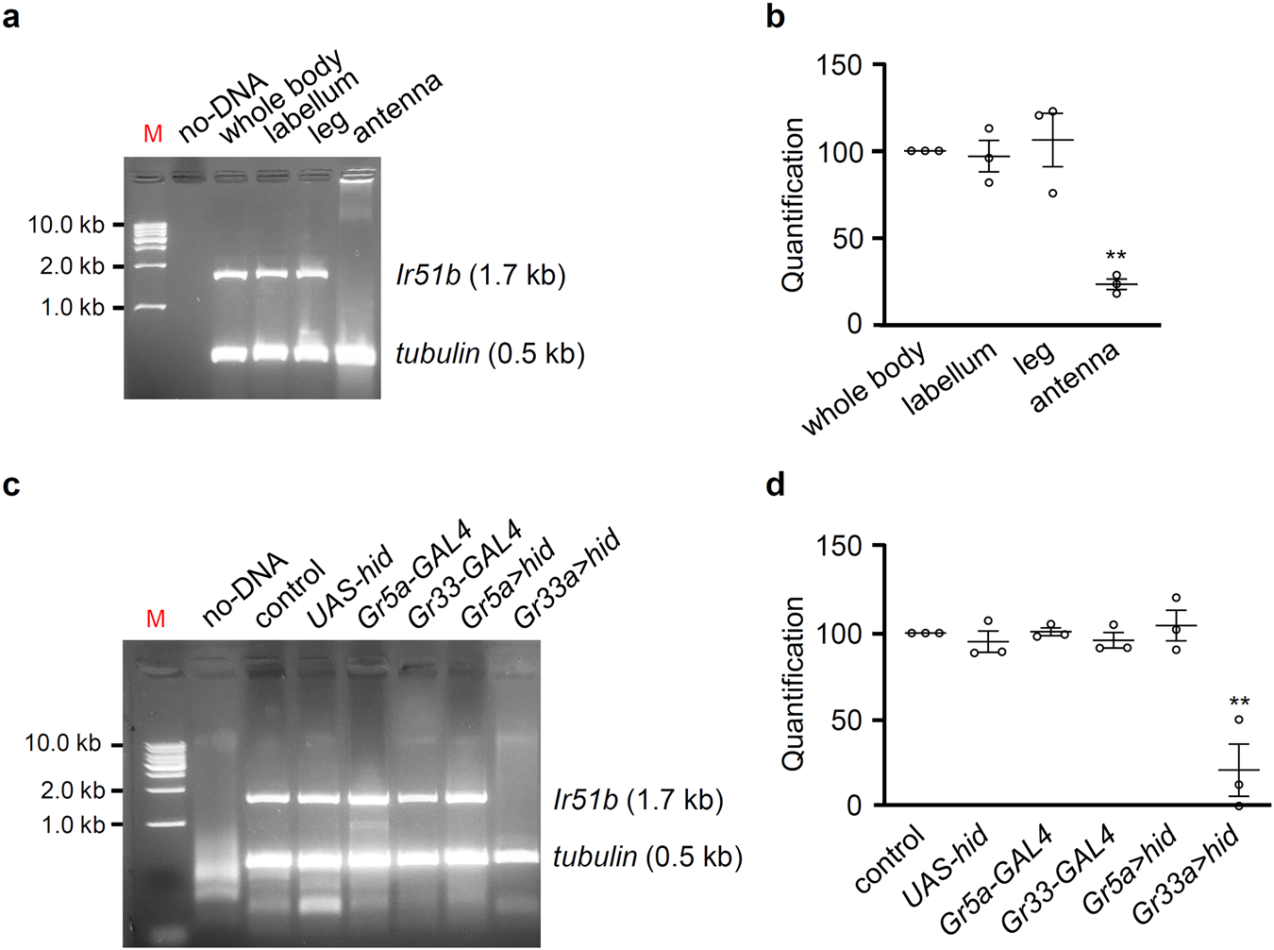
*Ir51b* is expressed in bitter-sensing GRNs on the labellum. **a** Gel picture of RT-PCR results showing *Ir51b* expression in the labellum and legs, but not in the antennae. Amplified *tubulin* products were used as control. “M” indicates the DNA ladder marker. **b** Quantification of *Ir51b* RNA levels which was normalized by *tubulin* in the same reaction in panel (**a**). The density of *Ir51b* RNA was divided by *tubulin* RNA level in each batch and control (whole body) was set to 100 by same fold change in the batch. Three repeated experiments were provided. **c** Gel picture of *Ir51b* expressed in bitter-sensing GRNs. The 1.7 kb *Ir51b* gene was amplified using RT-PCR in no-DNA template, the control (*w*^*1118*^), *UAS-hid*, *Gr5a-GAL4* (sugar-sensing GRNs), *Gr33a-GAL4* (bitter-sensing GRNs), *Gr5a*-*GAL4*/*UAS*-*hid* (sugar-sensing GRNs ablated), and *Gr33a*-*GAL4*/*UAS*-*hid* (bitter-sensing GRNs ablated) flies. Amplified *tubulin* products were used as internal control of PCR reaction. “M” indicates the DNA ladder marker. **d** Quantification of *Ir51b* RNA levels which was normalized by *tubulin* in the same reaction in panel (**c**). Three repeated experiments were provided. The density of *Ir51b* RNA was divided by *tubulin* RNA level in each batch and control (*w*^*1118*^) was set to 100 by same fold change in the batch. All error bars represent the SEMs. Multiple comparisons were conducted using single-factor ANOVA coupled with Scheffe’s post hoc test. The asterisks indicate statistical significance, compared to the control (whole-body sample in (**b**) and control in (**d**)) (^**^P < 0.01).

We previously reported that IR25a was co-expressed extensively with the IR76b reporter^29^. Further, a previous study indicated that the *Ir51b*-*GAL4* reporter was not detected in the labellum^15^. In addition, *Ir51b* is detected in the RNA-seq analysis of the Drosophila antenna^46^. Therefore, to investigate whether *Ir51b* RNA is expressed in the labellum, we performed reverse transcriptase-polymerase chain reaction (RT-PCR) using labellum, leg, and antenna samples (Fig. 4a and Supplementary Fig. 5a). We identified the predicted 1.7 kb band in the wild-type labella and legs, but not antennae. This band was confirmed by DNA sequencing. Furthermore, repeated experiments for three independent sample preparation were quantified by ImageJ and normalization (Fig. 4b). This suggests that IR51b acts as a contact-mediated chemosensor. However, we do not completely exclude any possible role of IR51b in antenna, because other group detects low-level expression of *Ir51b* by RNA-seq. Next, we expressed the cell death gene *UAS*-*hid* under the control of sweet-sensing (*Gr5a*-*GAL4*) or bitter-sensing (*Gr33a*-*GAL4*) receptors to detect whether *Ir51b* participates in the GRN-mediated perception of bitter substances using *tubulin* as an internal control (Fig. 4c, d and Supplementary Fig. 5b). We found that *Ir51b* RNA was almost completely eliminated in the *Gr33a*-*GAL4*/*UAS*-*hid* mutants (Fig. 4c, d and Supplementary Fig. 5b). Next, we generated a second *Ir51b* allele using ends-out homologous recombination (Supplementary Fig. 4). The in-frame knock-in of *GAL4* was generated with a 1017 bp deletion of the exon; however, we failed to recapitulate the expression pattern of *Ir51b*. Therefore, this mutant was named *Ir51b*^*2*^. This second allele also showed similar deficits to those of *Ir51b*^*1*^ (Fig. 2b–d), thus suggesting the role of IR51b in the GRN-mediated perception of bitter-tasting (aversive) compounds.

**Fig. 5:**
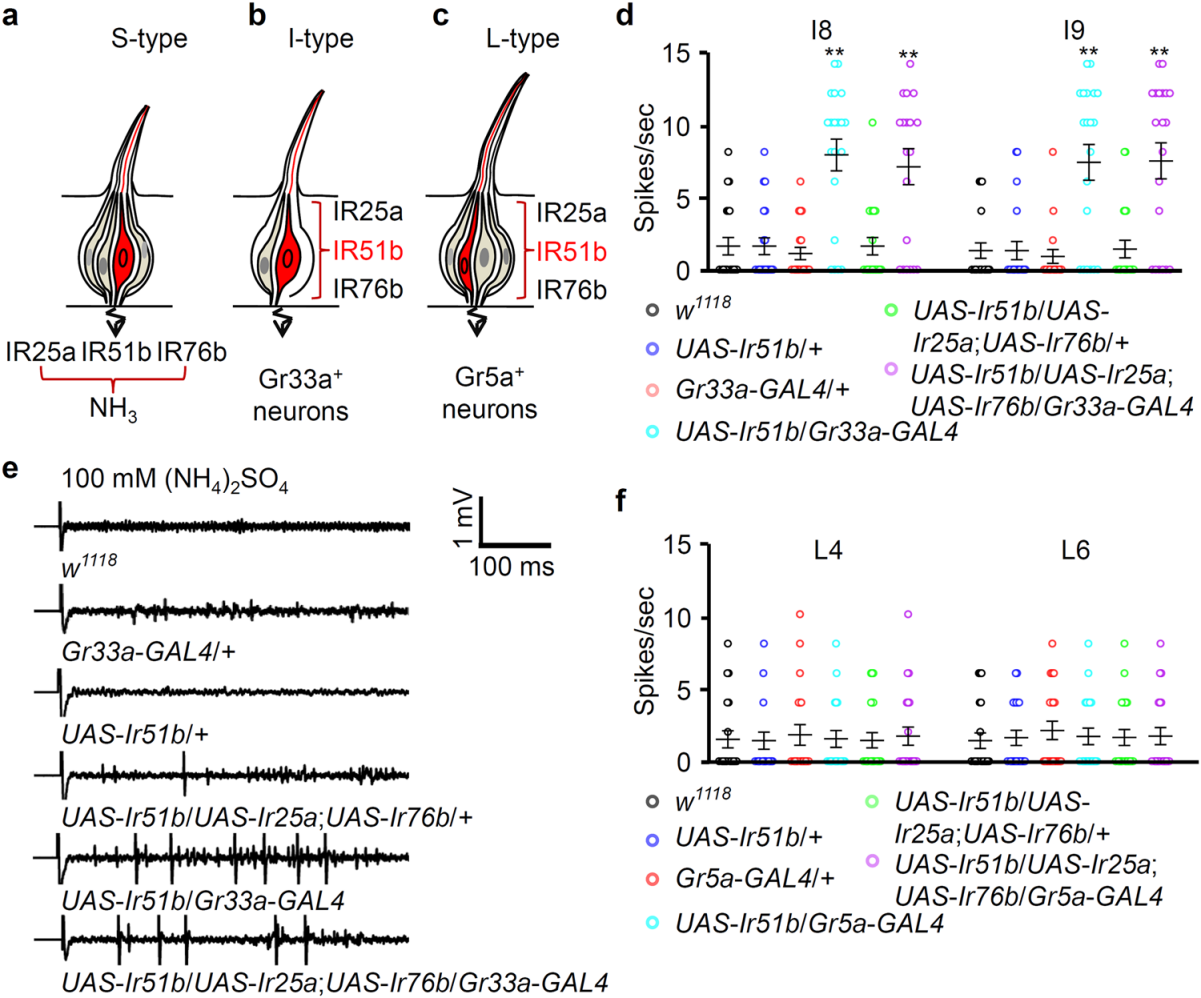
Overexpression of IR25a, IR51b, and IR76b in bitter-sensing or sugar-sensing GRNs. **a**–**c** Cartoons of three different types of gustatory sensilla and their GRNs on the labellum of Drosophila. **a** Hetero multimeric association of *Ir25a*, *Ir51b*, and *Ir76b* in S-type sensilla for ammonia taste processing. **b** Misexpression of *Ir51b* cDNA in I-type sensilla. **c** Misexpression of *Ir51b* cDNA in L-type sensilla. **d** *UAS*-*Ir51b* alone or *UAS*-*Ir25a*, *UAS*-*Ir51b, and UAS*-*Ir76b* were expressed in bitter-sensing GRNs under the control of *Gr33a*-*GAL4*. Average action potentials were generated on I8 and I9 sensilla from the labellum of the indicated genotypes with 100 mM (NH_4_)_2_SO_4_ (*n* = 20). **e** Representative sample traces from panel (**d**). **f** Overexpression of *Ir25a, Ir51b*, and *Ir76b* cDNA in sugar-sensing GRNs in L-type sensilla under the control of *Gr5a*-*GAL4*. Recordings of nerve responses were performed on L4 and L6 sensilla in the labellum of the indicated flies with 100 mM (NH_4_)_2_SO_4_ (*n* = 20). All error bars represent SEMs. Multiple comparisons were conducted using single-factor ANOVA coupled with Scheffe’s post hoc test (^**^P < 0.01).

To investigate the genetic recapitulation of ammonia-taste receptors, we assessed whether these three genes were sufficient to elicit taste-induced avoidance of nitrogenous compounds in flies. I-type sensilla in the fly’s labellum possess only two GRNs, whereas L-type and S-type sensilla harbor four GRNs (Fig. 5a–c). We then induced the expression of wild-type *Ir51b* cDNA in the bitter-sensing I-type cells or sweet-sensing L-type cells where they are normally not expressed based on our mapping results (Supplementary Fig. 1a). The action potentials of the I-type sensilla of flies carrying either the *UAS*-*Ir51b* or *Gr33a*-*GAL4* mutations were not significantly different from those of the *w*^*1118*^ strain. In contrast, ammonium sulfate elicited a significant increase in the I8 and I9 sensilla action potential frequency of *UAS*-*Ir51b*/*Gr33a*-*GAL4* flies (Fig. 5d, e). Furthermore, similar to our experiments where only the expression of Ir51b cDNA was induced, expression of all three cDNAs with *Gr33a-GAL4* elicited similar responses from I8 and I9 sensilla when presented with a 100 mM ammonium sulfate stimulus. These results were consistent with our previous report that IR25a and IR76b were involved in the GRN-mediated perception of bitter compounds^29^. Next, we expressed *UAS*-*Ir51b* only or with *UAS*-*Ir25a* and *UAS*-*Ir76b* under the control of the sweet-sensing *Gr5a*-*GAL4* and recorded from L-type sensilla (Fig. 5f). However, no significant differences from *w*^*1118*^ were observed. Therefore, our findings indicated that three IRs (IR25a, IR51b, and IR76b) were involved in the gustatory detection of nitrogenous waste products via heteromultimeric channel formation; however, ammonia sensing could not be recapitulated in sweet-sensing GRNs.

Overall, we identified three IRs to sense nitrogenous waste compounds in S-type. Furthermore, three IRs were enough to induce ammonium sulfate response in I-type sensilla. However, the combination of three IRs was insufficient to elicit physiological responses to ammonium sulfate in L-type sensilla when driven with *Gr5a*-*GAL4*. This indicates that additional channel subunits required for the responses to the nitrogenous waste compounds must be present in S-type and I-type GRNs. Therefore, this study has a limitation to claiming that the IRs are receptor ion channels for nitrogenous waste compounds, because we cannot rule out the function of IRs in transduction pathways. Future works should focus on finding additional channel subunits to prove that nitrogenous waste compounds can directly activate these IRs by heterologous expression.

Chemical sensation is an essential modulator of physiology and behavior. In invertebrates, the vast majority of chemical stimuli in the environment are recognized by members of two evolutionarily related chemosensory receptors: the ORs and the GRs^51^. However, recent studies have indicated that IRs are likely the most ancient chemoreceptors and thus predate ORs and GRs, as their existence can be traced back prior to the deuterostome-protostome split^16,27^.

Ammonia can act as a kairomone, and therefore some species, such as flour mites, are attracted to the microbial degradation products of certain amino acids^52^. Females of some uricotelic muscid flies are reportedly attracted to ammonia when searching for suitable oviposition sites^53^. In this case, ammonia acts as a chemical attractant that enables some parasitic organisms to detect their hosts. However, ammonia can also be used to kill or repel bed bugs, ants, rats, fleas, and snakes. Insect survival may also vary depending on ecological niche or host characteristics; however, the proliferation of insect populations is generally thought to be highly host-dependent. Here, we demonstrated that fruit flies avoided nitrogenous waste products both when laying eggs and when selecting their food, as these compounds are potentially toxic. Despite the differences in the mechanisms of chemical sensation between arthropods and humans, the identification of ammonia-associated taste sensors in insects provides important insights into how animals perceive and react to specific chemicals.

## Methods

### Fly strains

Unless otherwise indicated, all flies were maintained at 25 °C under a 12 h light/12 h dark cycle. Both male and female flies were used randomly in our experiments. The control strain used in this study was *w*^*1118*^.

We previously described the *Ir7a*^1^, *Ir47a*^1^*, Ir52a*^1^, *Ir56a*^*1*^, *Ir60b*^*3*^, *Ir94a*^*1*^, *Ir94c*^*1*^, and *Ir94h*^*1*^ strains^20^. The *Ir7g*^*1*^ (BL42420)*, Ir10a*^*1*^ (BL23842)*, Ir21a*^*1*^ *(BL17171), Ir48a*^*1*^ (BL26453)*, Ir48b*^*1*^ (BL23473)*, Ir51b*^*1*^ (BL10046)*, Ir52b*^*1*^ (BL25212)*, Ir56b*^*1*^ (BL27818)*, Ir62a*^*1*^ (BL32713)*, Ir67a*^*1*^ (BL56583)*, Ir75d*^*1*^ *(BL24205), Ir92a*^*1*^ *(BL58205), Ir94b*^*1*^ *(BL23424), Ir94d1* (BL33132)*, Ir94g*^*1*^ (BL25551), and *Ir100a*^*1*^ (BL31853). *Gr2a*^*1*^ (BL18415), *Gr10a*^*1*^ (BL29947), *Gr22f*^*1*^ (BL43859), *Gr23a*^*1*^ (BL19287), *Gr28b*^*Mi*^ (BL24190), *Gr36b*^*1*^ (BL24608), *Gr36c*^*1*^ (BL26496), *Gr58b*^*1*^ (BL29065), *Gr59a*^*1*^ (BL26125), *Gr77a*^*1*^ (BL26374), *Gr93d*^*1*^ (BL27800), *Gr94a*^*1*^ (BL17550), *Gr97a*^*1*^ (BL18949), *Ir8a*^*1*^ (BL41744), *Ir85a*^*1*^ (BL24590), *UAS*-*hid* (BL65403), and *UAS*-*Kir2.1* (BL6596*)* strains were obtained from the Bloomington Drosophila Stock Center. Additionally, we obtained the following mutants from the Korea Drosophila Resource Center: *Gr28a*^*1*^, *Gr36a*^*1*^, *Gr39b*^*1*^, *Gr59c*^*1*^*,* and *Gr89a*^*1*^. We previously described the *Gr8a*^*1*^ ^54^, *Gr33a*^*1*^ ^48^, *Gr47a*^*1*^ ^55^*, Gr66a*^*ex83*^ ^56^*, Gr33a*^*GAL4 48*^*, Gr98b*^*1*^ ^57^, *Ir76b*^*1*^ ^29^, *Ir76b*-*GAL4* ^29^, and *UAS-Ir76b* ^29^. K. Scott provided *ppk23*-*GAL4*^58^ and *ppk28*-*GAL4*^18^. H. Amrein gave *∆Gr32a*, *Gr66a*-*GAL4*, and *Gr5a*-*GAL4*^59,60^. L. Vosshall provided *Ir25a*^*2*^^23^. We obtained *Gr22e*^*1*^ (140936) from Kyoto Drosophila Stock Center.

### Chemical reagents

Sucrose (CAS No 57-50-1, Cat No S9378), sulforhodamine B (CAS No 3520-42-1, Cat No 230162), ammonium sulfate (CAS No 7783-20-2), ammonium chloride (CAS No 12125-02-9), putrescine (CAS No 333-93-7), and urea (CAS No 57-13-6) were purchased from Sigma-Aldrich Co. Brilliant blue FCF (CAS No 3844-45-9, Cat No 027-12842) was purchased from Wako Pure Chemical Industry Ltd.

### Generation of *Ir51b*^*2*^ mutant lines

The *Ir51b*^*2*^ knock-in-*GAL4* mutant line was created via ends-out homologous recombination as previously described^61^. Concretely, we amplified 2.94 kb upstream and 3.05 kb downstream of genomic fragments through PCR and subcloned the DNA into the pw35-GAL4 vector^48^. Right arm extension included 3050 bp and left arm extension included 2944 bp along with the ATG start codon. *GAL4* was inserted by replacing 1017 bp of genomic regions to preserve the reading frame of the ATG start codon. The construct was injected into *w*^*1118*^ embryos by the Korea Drosophila Resource Center (KDRC).

### RT-PCR

Labellum, leg, and antenna samples were dissected from approximately 30 control, *Gr5a*-*GAL4*/*UAS*-*hid*, and *Gr33a*-*GAL4*/*UAS*-*hid* adult flies. Total RNA was extracted using TRIzol (Invitrogen) and cDNA was synthesized using AMV reverse transcriptase (Promega). To perform the RT-PCR, we used the following *Ir51b* primers: 5′-GGC GCT AAC AAA CGC TGC TTAC -3′ and 5′-CAG AGC TGA CAG TAT CCA ACC AA-3′. The *tubulin* primers were 5′-TCC TTG TCG CGT GTG AAA CA-3′ and 5′-CCG AAC GAG TGG AAG ATG AG-3′. RT-PCR products were obtained after 35 cycles. Each samples were repeated at least three times. Intensity measurement was done by using ImageJ (Fiji) application and then *Ir51b* RNA level in each sample was normalized by the internal control, *tubulin*.

### Two-way food choice assay

To perform binary food choice assays, we followed a previously described protocol^29^. First, 5–7-day-old mixed gender (males and females were randomly selected) flies were starved in a vial containing water-soaked Kimwipe paper for 16–18 h in a dark and humid chamber. Each experiment was conducted using 50–70 flies. We then prepared two food options, both containing 1% agarose: one contained only sucrose and the other contained sucrose mixed with nitrogen-containing chemicals. These food sources were colored with either blue (brilliant blue FCF, 0.125 mg/mL) or red food-grade dye (sulforhodamine B, 0.1 mg/mL). These two food preparations were dispensed into a 72-well microtiter dish (Thermo Fisher Scientific, Cat No 438733) in an alternative position. We briefly anesthetized the starved flies and introduced them into the food dish, after which we immediately transferred them to an incubator for 90 min. The flies were euthanized in a −20 °C freezer for at least 2 h. Then, the abdomen color was classified as “blue,” “red,” or “purple” using a stereomicroscope. The preference index (PI) was calculated using following equations:

1 $${{{\mathrm{PI}}}} 	= (N_{{{\mathrm{red}}}}-N_{{{\mathrm{blue}}}})/(N_{{{\mathrm{red}}}} + N_{{{\mathrm{blue}}}} + N_{{{\mathrm{purple}}}}), {{{\mbox{or}}}}\\ {{{\mathrm{PI}}}} 	= \left[\right.(N_{{{\mathrm{blue}}}} - N_{{{\mathrm{red}}}})/(N_{{{\mathrm{red}}}} + N_{{{\mathrm{blue}}}} + N_{{{\mathrm{purple}}}})$$

depending on the dye/tastant combinations. At least six replicates were performed for each fly strain.

### Electrophysiology

Tip recording assays were conducted as described in a previous study^29^. First, 4–6-day-old flies were anesthetized on ice. A reference glass electrode filled with Ringer’s solution (3 mM CaCl_2_, 182 mM KCl, 46 mM NaCl, and 10 mM Tris-base; pH 7.2) was inserted into the thorax of the flies. The glass electrode was gently pushed towards their proboscis without causing any severe damage to the GRNs on the proboscis. Approximately 4–6 flies were used for each experiment. Using an electrophysiology system, we activated the S-type, I-type, and L-type taste sensilla on the labella of flies for 5 s using a mixture of tastants with 30 mM tricholine citrate (TCC). The recording electrode (10–20 μm tip diameter) was connected to a preamplifier (Taste PROBE, Syntech, Hilversum, The Netherlands), and the signals were collected and amplified by 10x using a signal connection interface box (Syntech) in conjunction with a 100–3000 Hz band-pass filter. Recordings of action potentials were acquired using a 12 kHz sampling rate and analyzed using the Autospike 3.1 software (Syntech). We then counted the action potentials for 50–550 ms and presented doubled values of the period per second in the figures. Each consecutive recording was performed with an approximately 1 min gap between each stimulation. The sample numbers (*n*) in each experiment indicate the number of animals. The same procedure was repeated on different days and using different setups.

### Proboscis extension reflex (PER) assay

The PER assay was performed as previously described^62^. The flies were first starved for 20–24 h in a vial with water-soaked Kimwipe paper. The flies were then briefly anesthetized on ice and fixed on a glass slide using glue. A fine Kimwipe paper wick was then used to deliver the initial 100 mM sucrose stimulus to the flies. Only flies that showed a positive PER to sucrose were considered for the next test. Taste stimuli were delivered to the labellum at least three times to avoid false-positive responses. At this point, only the flies that exhibited a positive PER to the experimental solutions (i.e., 100 mM ammonia in 100 mM sucrose) were deemed PER positive. A total of 10–15 flies were evaluated per experiment, after which PER percentages were calculated. At least six replicates were performed for each strain.

### Oviposition preference assay

Oviposition preference assays were conducted as described in a previous study^63^. A total of 15 female and 15 male newly hatched flies were transferred into a new food vial supplemented with dry yeast and kept in a normal light/dark cycle for two days. Prior to the assays, the experimental animals were acclimatized in 1% agarose containing a test food choice for 5–6 h. Two food options were then provided, one containing only sucrose and another containing a mixture of sucrose and a nitrogen-containing chemical, both of which were dispensed on a Petri dish (35 mm diameter, Product No. 351007) divided into two equal halves. The agarose food was allowed to solidify and then transferred to an egg-laying chamber (Code No. FEC-50200, Hansol Tech, Republic of Korea); the acclimated flies were transferred into the chamber thereafter. The flies were then allowed to lay eggs overnight inside of the incubator. The next day, the number of eggs deposited on each side of the chamber (i.e., each containing a different food option) was counted, and the ovipositional preference index was calculated as follows:

2 $$	({{{\mathrm{number}}}}\; {{{\mathrm{of}}}}\; {{{\mathrm{eggs}}}}\; {{{\mathrm{laid}}}}\; {{{\mathrm{on}}}}\; {{{\mathrm{the}}}}\; {{{\mathrm{control}}}}\; {{{\mathrm{plate}}}}) \\ 	\quad- ({{{\mathrm{number}}}}\; {{{\mathrm{of}}}}\; {{{\mathrm{eggs}}}}\; {{{\mathrm{laid}}}}\; {{{\mathrm{on}}}}\; {{{\mathrm{the}}}}\; {{{\mathrm{chemical}}}}{\mbox{-}}{{{\mathrm{containing}}}}\; {{{\mathrm{plate}}}})/\\ 	\quad{{{\mathrm{total}}}}\; {{{\mathrm{number}}}}\; {{{\mathrm{of}}}}\; {{{\mathrm{laid}}}}\; {{{\mathrm{eggs}}}}.$$

At least six replicates were performed for each strain.

### Statistics and reproducibility

We selected *D.* *melanogaster* as a model animal. Both sexes of experimental animals were considered randomly for the experiments we performed. All the experiments were conducted at laboratory conditions. Based on the previous studies, we determined at least six replicates per genotype were enough to verify behavioral data, where as at least 10 animals per genotype were enough in electrophysiological recordings. We performed three replicates to analyze *Ir51b* expression for RNAi analysis. We met enough sample size to make our data more reliable in each figure. Each experiment was conducted for at least two different days. No data was excluded from the analysis. Each data points represents a real value. Average of all the replicates for that specific genotypes were presented. All error bars represent the standard error of the mean (SEM). Multiple comparisons were then evaluated using single-factor ANOVA coupled with Scheffe’s post hoc test. Asterisks indicate statistical significance (^*^P < 0.05, ^**^P < 0.01). Statistical analyses were performed using Origin Pro 8 for Windows (ver. 8.0932; Origin Lab Corporation, USA).

### Reporting summary

Further information on research design is available in the Nature Research Reporting Summary linked to this article.

## Supplementary information

Supplementary information.

Description of Additional Supplementary Files.

Supplementary Data 1.

Reporting summary.

## Data Availability

The datasets used in this paper are preserved with corresponding author, which are available upon resonable request. The source data for the individual values and scripts used to generate figures are attached to this paper as Supplementary Data 1.
