# Supplementary material for: Ionotropic receptors mediate nitrogenous waste avoidance in Drosophila melanogaster
Source: Commun Biol. 2021 Nov 12;4:1281. doi: 10.1038/s42003-021-02799-3 (PMC8589963; doi:10.1038/s42003-021-02799-3)
Supplement: Supplementary file 1 — Supplementary information. [file 42003_2021_2799_MOESM1_ESM.pdf]

Supplementary Information

Ionotropic receptors mediate nitrogenous waste avoidance in *Drosophila melanogaster*

Subash Dhakal[1], Jiun Sang[1], Binod Aryal[1], and Youngseok Lee[1,2*]

[1]Department of Bio and Fermentation Convergence Technology, Kookmin University, Seoul, 02707, Republic of Korea

[2]Interdisciplinary Program for Bio-Health Convergence, Kookmin University, Seoul 02707, Republic of Korea

*Correspondence and requests for materials should be addressed to:

Y.L. (email: ylee@kookmin.ac.kr; Orchid: 0000 0003 0459 1138)

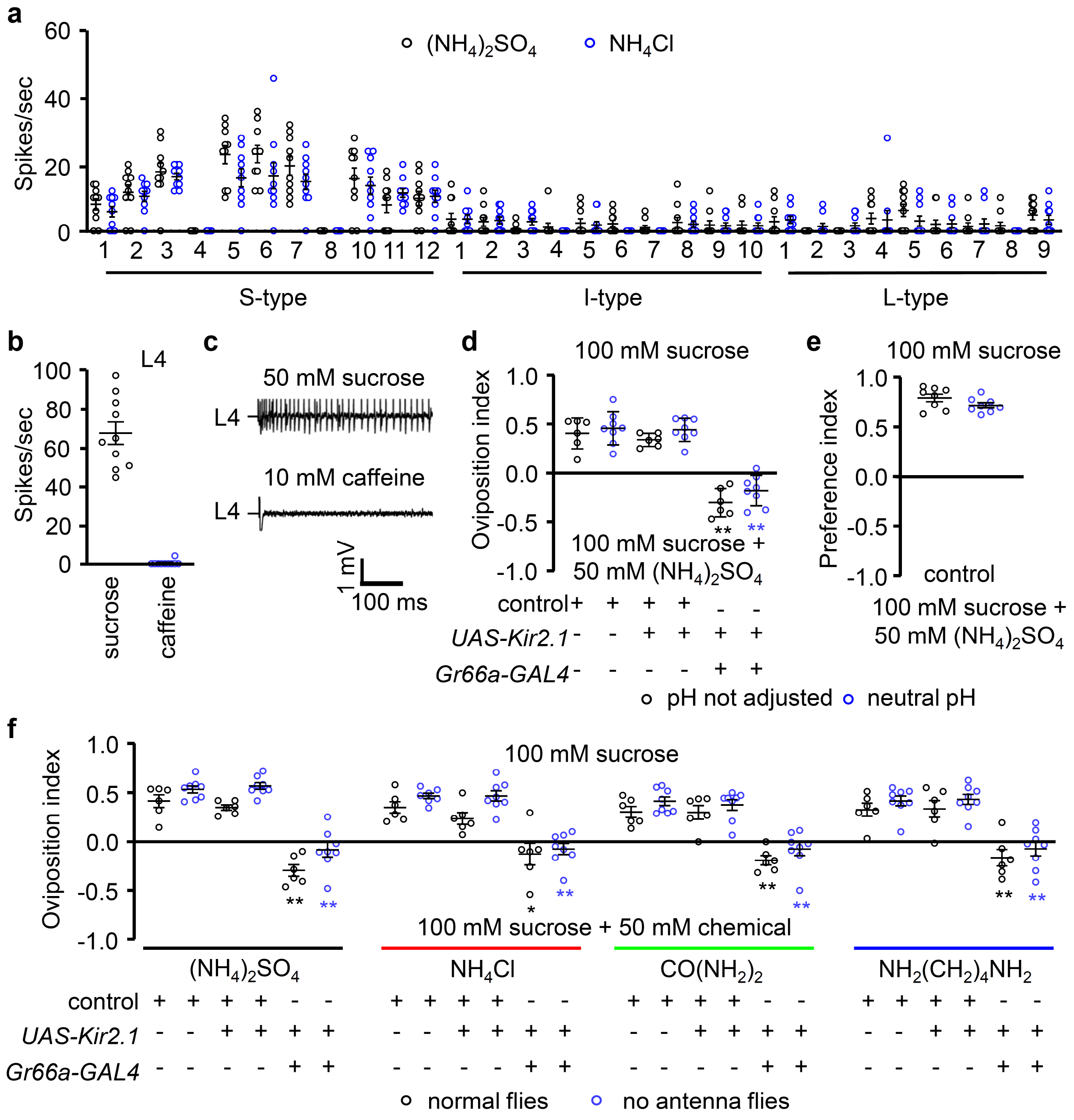

Supplementary Figure. 1. Flies avoid ammonia in behavior and physiology

a Averages of action potentials produced against 100 mM ammonium sulfate [$(NH_4)_2SO_4$] and ammonium chloride (NH_4Cl) from all 31 taste sensilla on the labellum of control (*w^{1118}*) flies (n=10).

b Averages of action potentials produced against 50 mM sucrose (black dots) and 10 mM caffeine (blue dots) as positive and negative control on L4 sensilla, respectively (n=10).

c Representative sample traces from **b.**

d Ovipositional preference assay of control (w^{1118}), *UAS-Kir2.1*/+, and bitter-sensing GRN-ablated flies (*Gr66a-GAL4*/*UAS-Kir2.1*) between 100 mM sucrose food and food containing 100 mM sucrose laced with 50 mM $(NH_4)_2SO_4$. Black dots indicate pH not adjusted (pH 5.8) and blue dots indicate pH adjusted (pH 7.0, neutral) condition, respectively (n=6-8).

e Binary food choice preference assay of control (w^{1118}) flies with 100 mM sucrose vs 100 mM sucrose mixed with 50 mM $(NH_4)_2SO_4$. Black and blue dots indicate preference index in pH not adjusted and pH adjusted (neutral pH) conditions, respectively (n=8).

f Female oviposition assay of control (w^{1118}), *UAS-Kir2.1*/+, and bitter-sensing GRN-ablated flies (*Gr66a-GAL4*/*UAS-Kir2.1*) between 100 mM sucrose food and food containing 100 mM sucrose laced with 50 mM $(NH_4)_2SO_4$, NH4Cl, $CO(NH_2)_2$, and $NH_2(CH_2)_4NH_2$ with normal and surgically antenna removed flies. Black dots indicate flies with antenna whereas blue dots indicate no antenna flies (n=6-8).

All error bars represent SEMs. Single factor ANOVA with Scheffe's analysis was used as a *post hoc* test to compare multiple sets of data ($^{**}P < 0.01$, $^{*}P < 0.05$). In Supplementary Fig. 1**d** and 1**f** black or blue color asterisks indicate statistical comparison within respective colored genotypes.

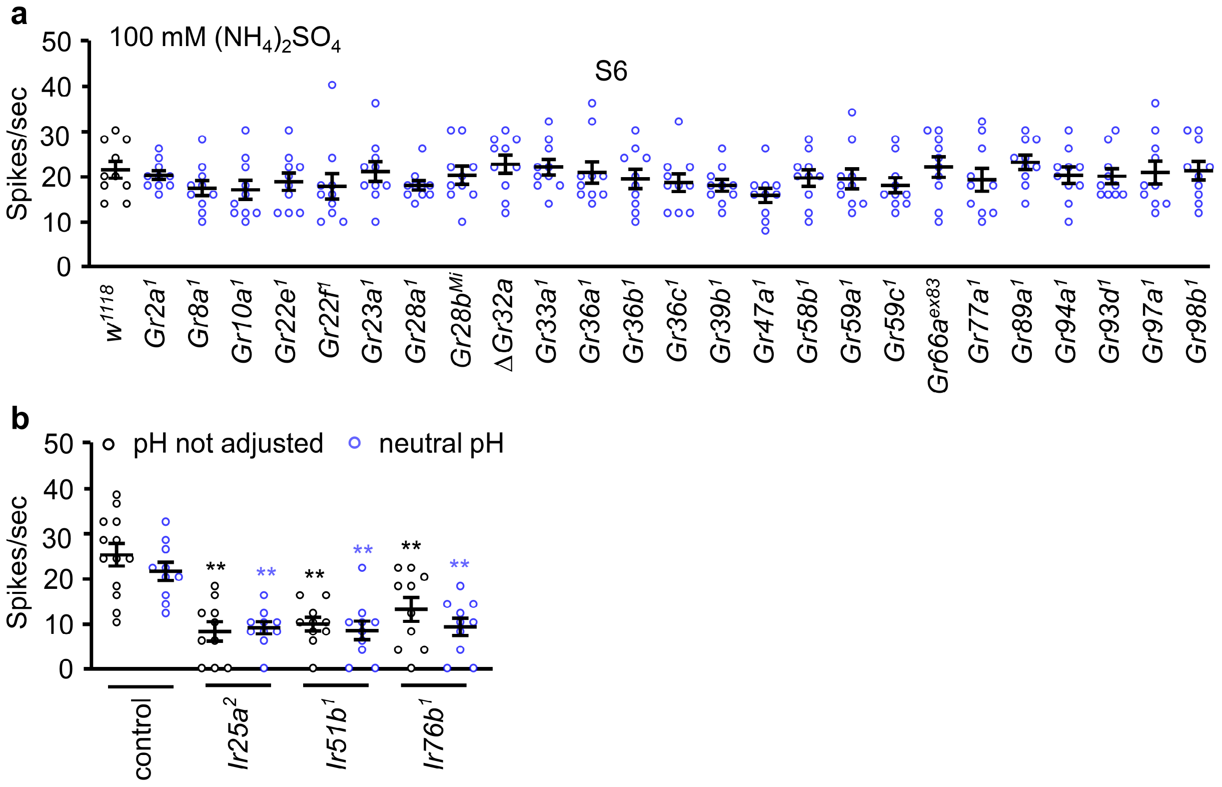

Supplementary Figure. 2. Tip recording assay of *Grs* mutants and candidate *Ir* mutants

a Responses of control (w^{1118}) and 26 Gustatory receptor (*Gr*) mutants to 100 mM $(NH_4)_2SO_4$ from S6 sensilla (n=10).

b Tip recording response of control (w^{1118}), *Ir25a*^{2}*, Ir51b*^{1}*,* and *Ir76b*^{1} mutants with 100 mM $(NH_4)_2SO_4$ in pH not adjusted (pH 5.8) and pH adjusted (pH 7.0, neutral) (n=10). All error bars represent SEMs. Single factor ANOVA with Scheffe's analysis was used as a *post hoc* test to compare multiple sets of data ($^{**}P < 0.01$). In Supplementary Fig. 2**b** black or blue color asterisks indicate statistical comparison within respective colored genotypes.

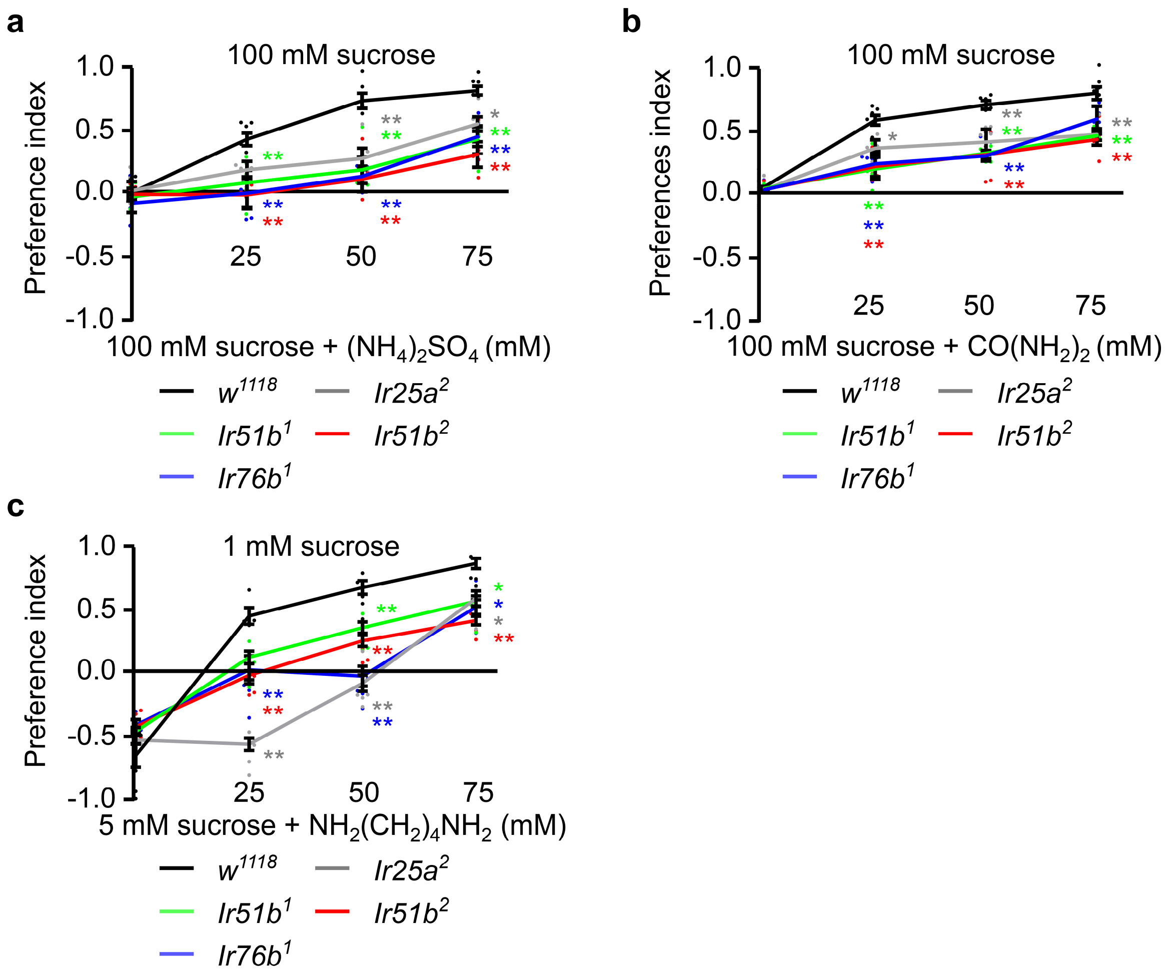

Supplementary Figure. 3. IRs present in the fly taste sensilla mediates ammonia taste

a—c Binary food choice assay of control (*w*[1118]), *Ir25a*[2], *Ir51b*[1], *Ir51b*[2], and *Ir76b*[1] flies. One food option is sucrose (mM) only and the other food contains sucrose (mM) and the indicated concentration of chemical, respectively.

a 100 mM sucrose versus 100 mM sucrose and 50 mM $(NH_4)_2SO_4$ (n=6).

b 100 mM sucrose versus 100 mM sucrose and 50 mM $CO(NH_2)_2$ (n=6).

c 1 mM sucrose versus 5 mM sucrose and 50 mM $NH_2(CH_2)_4NH_2$ (n=6).

All error bars represent SEMs. Single factor ANOVA with Scheffe's analysis was used as a *post hoc* test to compare multiple sets of data ($^{**}P < 0.01$, $^{*}P < 0.05$). Each color asterisks indicate statistical comparison within respective colored genotypes.

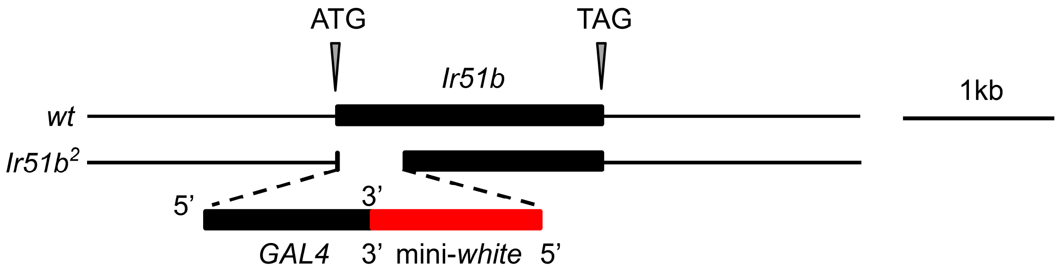

Supplementary Figure. 4. *Ir51b* knock-in mutant gene structure

Gene structure of the *Ir51b*^{2} knock-in *GAL4* mutant. Knock-in *GAL4* mutant generation via homologous recombination. *GAL4* was inserted into the *Ir51b* locus in-frame.

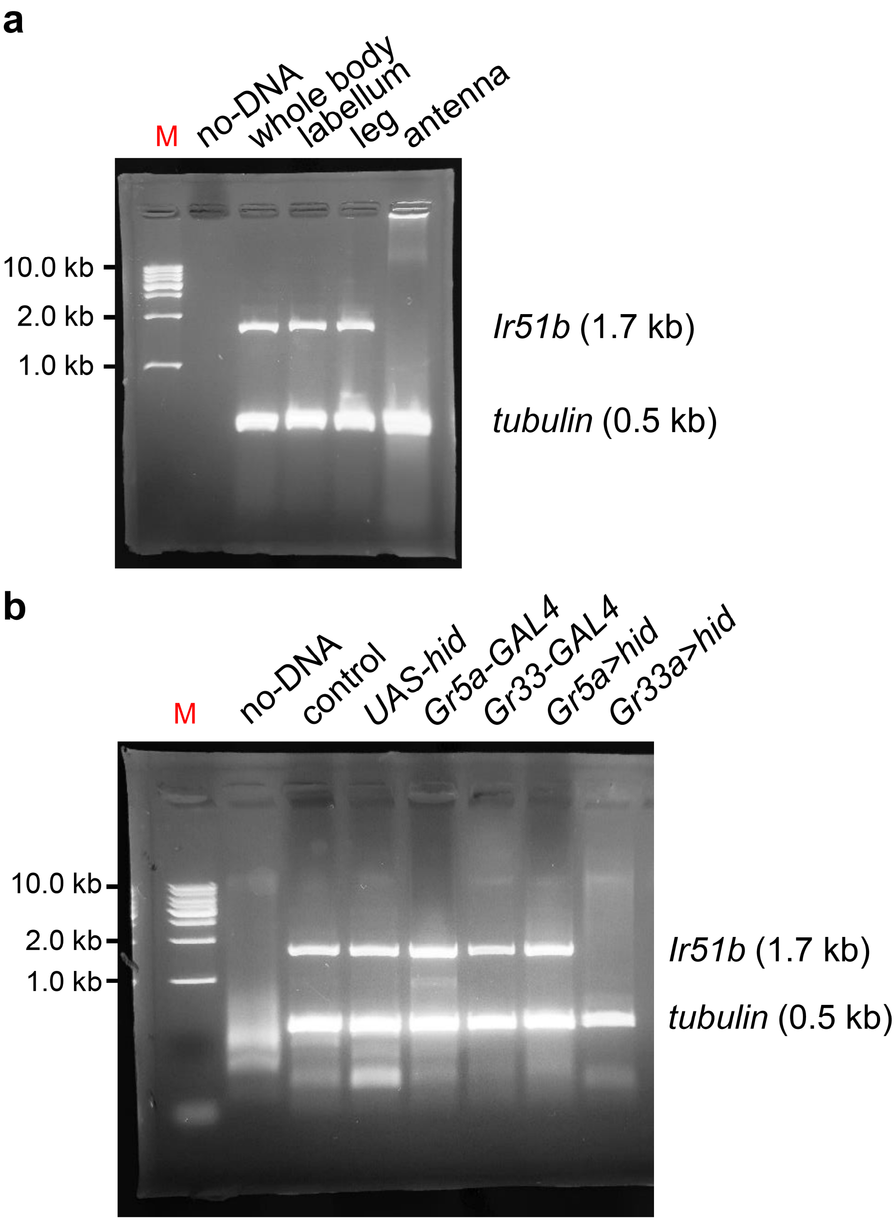

Supplementary Figure. 5. Gel picture of *Ir51b* expression by RT-PCR

Original gel picture of Figure 4a and 4c.

Genotype	Stock #	Mutation type	Reference
Ir7a^{1}	BL81940	Deletion/Insertion	1
Ir7g^{1}	BL42420	Insertion	2
Ir8a^{1}	BL41744	Deletion	3
Ir10a^{1}	BL23842	Deletion	4
Ir21a^{1}	BL10975	Insertion	5
Ir25a^{2}	BL27789	Deletion/Insertion	6
Ir47a^{1}	N/A	Deletion/Insertion	1
Ir48a^{1}	BL26453	Insertion	4
Ir48b^{1}	BL23473	Insertion	This paper
Ir51b^{1}	BL10046	Insertion	7
Ir52a^{1}	N/A	Deletion/Insertion	1
Ir52b^{1}	BL25212	Insertion	4
Ir56a^{1}	N/A	Deletion/Insertion	1
Ir56b^{1}	BL27818	Insertion	4
Ir60b^{3}	N/A	Deletion/Insertion	1
Ir62a^{1}	BL32713	Insertion	2
Ir67a^{1}	BL56583	Insertion	2
Ir75d^{1}	BL24205	Insertion	4
Ir76b^{1}	BL51309	Insertion	8
Ir85a^{1}	BL24590	Insertion	4
Ir92a^{1}	BL23638	Insertion	4
Ir94a^{1}	N/A	Deletion/Insertion	1
Ir94b^{1}	BL23424	Insertion	4
Ir94c^{1}	N/A	Deletion/Insertion	1
Ir94d^{1}	BL33132	Insertion	2
Ir94g^{1}	BL25551	Insertion	4
Ir94h^{1}	N/A	Deletion/Insertion	1
Ir100a^{1}	BL31853	Insertion	4
Gr2a^{1}	BL18415	Insertion	5
Gr8a^{1}	BL40976	Deletion	9
Gr10a^{1}	BL29947	Insertion	4
Gr22e^{1}	DGRC140936	Insertion	10
Gr22f^{1}	BL43859	Insertion	2
Gr23a^{1}	BL19287	Insertion	5
Gr28a^{1}	KDRC10041	Insertion	11
Gr28b^{Mi}	BL24190	Insertion	4
Δ*Gr32a*	N/A	Deletion/Insertion	12
Gr33a^{1}	BL31427	Deletion/Insertion	13
Gr36a^{1}	KDRC10042	Insertion	11
Gr36b^{1}	BL24608	Insertion	4
Gr36c^{1}	BL26496	Insertion	4
Gr39b^{1}	KDRC10043	Insertion	11

Gr47a^{1}	BL65843	Deletion	14
Gr58b^{1}	BL29065	Insertion	4
Gr59a^{1}	BL26125	Insertion	4
Gr59c^{1}	KDRC10044	Insertion	11
Gr66a^{ex83}	BL25027	Deletion	14
Gr77a^{1}	BL26374	Insertion	4
Gr89a^{1}	KDRC10045	Insertion	11
Gr93d^{1}	BL27800	Insertion	4
Gr94a^{1}	BL17550	Insertion	15
Gr97a^{1}	BL18949	Insertion	5
Gr98b^{1}	N/A	Deletion	16

Supplementary Table 1. Nature of mutation in *Ir* and *Gr* and mutants

List of *Ir* and *Gr* mutants we used in our study. This table provides the information about types of mutations done to generate each mutant line.

Supplementary References

1 Rimal, S. *et al.* Mechanism of acetic acid gustatory repulsion in Drosophila. *Cell reports* **26**, 1432-1442. e1434 (2019).

2 Nagarkar-Jaiswal, S. *et al.* A library of MiMICs allows tagging of genes and reversible, spatial and temporal knockdown of proteins in Drosophila. *elife* **4**, e05338 (2015).

3 Abuin, L. *et al.* Functional architecture of olfactory ionotropic glutamate receptors. *Neuron* **69**, 44-60 (2011).

4 Bellen, H. J. *et al.* The Drosophila gene disruption project: progress using transposons with distinctive site specificities. *Genetics* **188**, 731-743 (2011).

5 Thibault, S. T. *et al.* A complementary transposon tool kit for Drosophila melanogaster using P and piggyBac. *Nature genetics* **36**, 283-287 (2004).

6 Benton, R., Vannice, K. S., Gomez-Diaz, C. & Vosshall, L. B. Variant ionotropic glutamate receptors as chemosensory receptors in Drosophila. *Cell* **136**, 149-162 (2009).

7 Rimal, S., Sang, J., Dhakal, S. & Lee, Y. Cucurbitacin B activates Bitter-Sensing gustatory receptor neurons via gustatory receptor 33a in Drosophila melanogaster. *Molecules and Cells* **43**, 530 (2020).

8 Zhang, Y. V., Ni, J. & Montell, C. The molecular basis for attractive salt-taste coding in Drosophila. *Science* **340**, 1334-1338 (2013).

9 Lee, Y. *et al.* Gustatory receptors required for avoiding the insecticide L-canavanine. *Journal of Neuroscience* **32**, 1429-1435 (2012).

10 Poudel, S., Kim, Y., Gwak, J.-S., Jeong, S. & Lee, Y. Gustatory receptor 22e is essential for sensing chloroquine and strychnine in Drosophila melanogaster. *Insect biochemistry and molecular biology* **88**, 30-36 (2017).

11 Sung, H. Y. *et al.* Heterogeneity in the Drosophila gustatory receptor complexes that detect aversive compounds. *Nature communications* **8**, 1-10 (2017).

12 Miyamoto, T. & Amrein, H. Suppression of male courtship by a Drosophila pheromone receptor. *Nature neuroscience* **11**, 874-876 (2008).

13 Moon, S. J., Lee, Y., Jiao, Y. & Montell, C. A Drosophila gustatory receptor essential for aversive taste and inhibiting male-to-male courtship. *Current Biology* **19**, 1623-1627 (2009).

14 Lee, Y., Moon, S. J., Wang, Y. & Montell, C. A Drosophila gustatory receptor required for strychnine sensation. *Chemical senses* **40**, 525-533 (2015).

15 Bellen, H. J. *et al.* The BDGP gene disruption project: single transposon insertions associated with 40% of Drosophila genes. *Genetics* **167**, 761-781 (2004).

16 Shim, J. *et al.* The full repertoire of Drosophila gustatory receptors for detecting an aversive compound. *Nature communications* **6**, 1-8 (2015).
