# Supplementary material for: Ionotropic receptors mediate nitrogenous waste avoidance in Drosophila melanogaster
Source: Commun Biol. 2021 Nov 12;4:1281. doi: 10.1038/s42003-021-02799-3 (PMC8589963; doi:10.1038/s42003-021-02799-3)
Supplement: Supplementary file 2 — Description of Additional Supplementary Files. [file 42003_2021_2799_MOESM2_ESM.pdf]

Description of Additional Supplementary Files

File name: Supplementary Data 1

Description: Source data of the graphs in the main figures (1-5) and supplementary figures (1-3). Data (numerical values) obtained from behavioral assays, electrophysiological assays, and PCR analysis were used to generate different figures.
